# Improved Task Performance, Low Workload, and User-Centered Design in Medical Diagnostic Equipment Enhance Decision Confidence of Anesthesia Providers: A Meta-Analysis and a Multicenter Online Survey

**DOI:** 10.3390/diagnostics12081835

**Published:** 2022-07-29

**Authors:** Alexandra D. Budowski, Lisa Bergauer, Clara Castellucci, Julia Braun, Christoph B. Nöthiger, Donat R. Spahn, David W. Tscholl, Tadzio R. Roche

**Affiliations:** 1 Department of Anesthesiology, University of Zurich, University Hospital Zurich, 8091 Zurich, Switzerland; alexandra.budowski@usz.ch (A.D.B.); lisa.bergauer@gmx.de (L.B.); clara.castellucci@usz.ch (C.C.); christoph.noethiger@usz.ch (C.B.N.); donat.spahn@usz.ch (D.R.S.); david.tscholl@usz.ch (D.W.T.); 2 Department of Epidemiology and Biostatistics, University of Zurich, 8006 Zurich, Switzerland; julia.braun-gruebel@uzh.ch

**Keywords:** diagnostic confidence, diagnostic, gender, over-confidence, self-assessment, online survey, under-confidence, user-centered design, workload

## Abstract

Decision confidence—the subjective belief to have made the right decision—is central in planning actions in a complex environment such as the medical field. It is unclear by which factors it is influenced. We analyzed a pooled data set of eight studies and performed a multicenter online survey assessing anesthesiologists’ opinions on decision confidence. By applying mixed models and using multiple imputation to determine the effect of missing values from the dataset on the results, we investigated how task performance, perceived workload, the utilization of user-centered medical diagnostic devices, job, work experience, and gender affected decision confidence. The odds of being confident increased with better task performance (OR: 1.27, 95% CI: 0.94 to 1.7; *p* = 0.12; after multiple imputation OR: 3.19, 95% CI: 2.29 to 4.45; *p* < 0.001) and when user-centered medical devices were used (OR: 5.01, 95% CI: 3.67 to 6.85; *p* < 0.001; after multiple imputation OR: 3.58, 95% CI: 2.65 to 4.85; *p* < 0.001). The odds of being confident decreased with higher perceived workload (OR: 0.94, 95% CI: 0.93 to 0.95; *p* < 0.001; after multiple imputation, OR: 0.94, 95% CI: 0.93 to 0.95; *p* < 0.001). Other factors, such as gender, job, or professional experience, did not affect decision confidence. Most anesthesiologists who participated in the online survey agreed that task performance (25 of 30; 83%), perceived workload (24 of 30; 80%), work experience (28 of 30; 93%), and job (21 of 30; 70%) influence decision confidence. Improved task performance, lower perceived workload, and user-centered design in medical equipment enhanced the decision confidence of anesthesia providers.

## 1. Introduction

Decision confidence is the degree to which an individual believes that his or her decision is likely to be correct [1,2]. In complex environments such as the medical field, estimating one’s confidence is key in planning further actions, as a decision can consequently impact a patient’s outcome [3,4,5]. For example, the decision of the surgeon to perform a high-risk procedure depends partly on how confident he or she is that the underlying diagnosis is correct and how confident he or she is in their surgical skills. Furthermore, decision confidence influences learning, searching for information, and decision-making optimization [2,6].

Despite the importance, it is unclear which factors influence and lead to higher or lower decision confidence. Considering that the successful completion of a task should lead to improved confidence in the work field, it is surprising that studies have shown only a weak positive correlation [7,8,9]. Similarly, the effect of a higher work experience showed contradictory results on decision confidence [7,10,11]. Another topic that has received much attention is over- and under-confidence [12]. Over-confident physicians are prone to diagnostic inaccuracies and suboptimal treatment [13,14]. Further, whether there is a gender difference as described in psychology is still unclear [7,12,15].

Due to the partly inconsistent and weak data, we conducted a pooled-data analysis and an online survey to identify factors affecting the decision confidence of anesthesia providers. More specifically, we analyzed whether task performance, perceived workload, the utilization of user-centered medical diagnostic devices, job (staff physician, resident physician, anesthesia nurse, or medical student), and work experience affected decision confidence. Additionally, we investigated the influence of gender on over- and under-confidence.

## 2. Materials and Methods

Before conducting the individual studies of this pooled-data analysis, we received permission from the responsible local ethic committees in Zurich, Frankfurt, Wuerzburg, Buenos Aires, and Barcelona, and each reviewed the different study protocols and issued a declaration of no objection. Additionally, all participants agreed to the analysis and publication of their data in written form.

### 2.1. Studies for Pooled-Data Analysis

**Figure 1:**
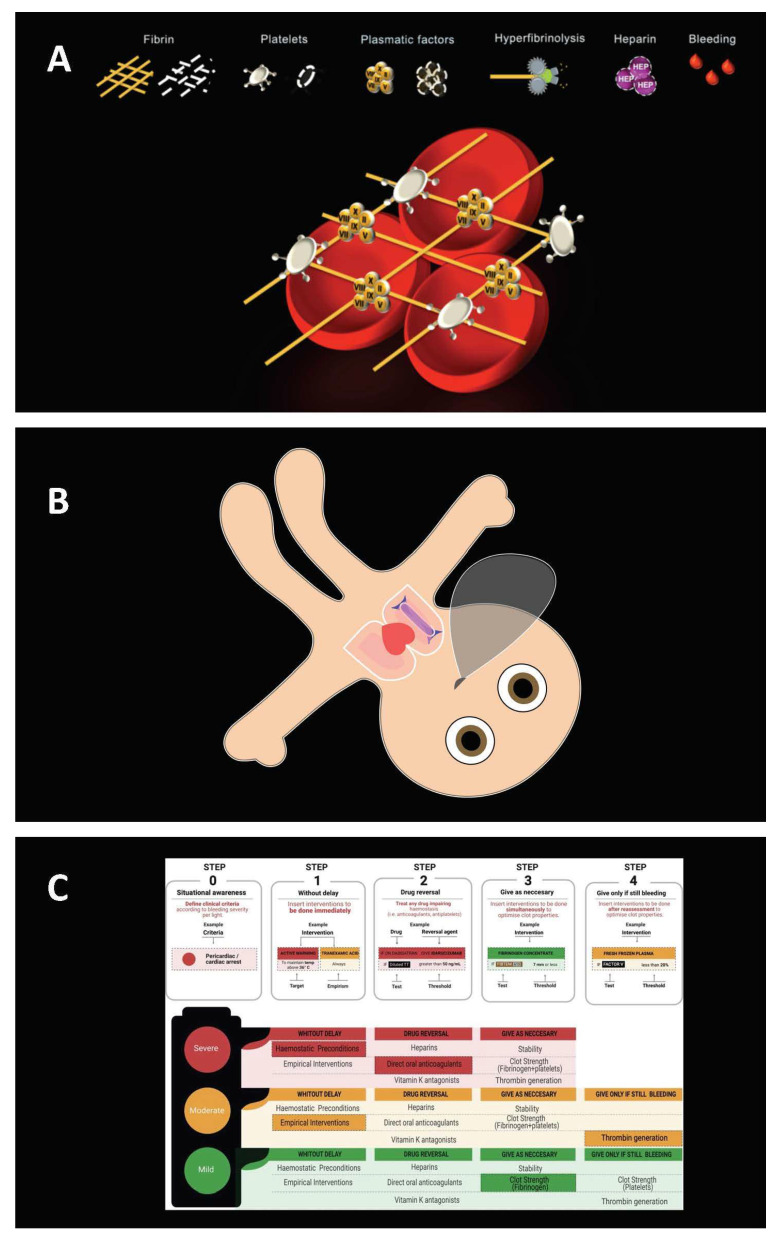
Presentation of the user-centered technologies. All were developed according to user-centered design principles. (**A**) A Visual Clot without pathologies is seen in the lower part of the image. The schematic icons displayed above represent the basic hemostatic components. For example, in the case of deficiency, components appear as dashed lines. (**B**) A Visual Patient with a high brain-activity index is represented by open eyes. The remaining vital signs are within the normal range. (**C**) Step-by-step design of Haemostasis Traffic Light. The cognitive aid can be adapted to local bleeding management protocols.

Our study group published eight researcher-initiated, prospective studies. Each study compared a new user-centered diagnostic technology with a conventional and established one used in anesthesia departments. Figure 1 illustrates the new user-centered diagnostic technologies. The self-assessment of decision confidence in solving a particular task correctly was an essential part of all study protocols. While participants utilized user-centered or conventional technology, they had to make diagnostic or therapeutic decisions. Thereafter, we asked them how confident they were that their decision was correct (decision confidence). In addition, we measured whether the task was solved correctly or incorrectly (task performance) and whether the task was solved using user-centered or conventional technology. Furthermore, we collected demographic data such as gender, job (staff physician, resident physician, anesthesia nurse, or medical student), and work experience. In all but one study, we also measured the participants’ perceived workload using the National Aeronautics and Space Administration Task Load Index (NASA TLX) [16]. Table 1 provides an overview of the studies included and their basic characteristics [17,18,19,20,21,22,23,24].

### 2.2. Investigated User-Centered Diagnostic Technologies

The following section and Figure 1 are intended to provide a deeper understanding of the user-centered technologies used in the studies. Following the ideas of Mica Endsley on situation awareness-oriented design [25], each technology was developed by incorporating user-centered design principles to facilitate the user’s work and to enhance the situational awareness of the medical professional.

Visual Patient Avatar is an innovative patient monitoring technology that visualizes numerical and waveform data into a picture—the patient avatar. This tool displays vital signs and possible vital-sign changes in real-time by adjusting the avatar’s shape, color, icons, and rhythmic pulsations. For example, open eyes represent a high brain-activity index, while purple skin color indicates low oxygen saturation [17,18,19,22]. Video S1 in the Supplementary Materials shows the Visual Patient Avatar and its various vital sign changes.

Visual Clot is an alternative visualization of rotational thromboelastometry (ROTEM) results, a viscoelastic coagulation test that allows the assessment of hemostasis from clot formation to clot dissolution. An algorithm transcribes existing parameters from the ROTEM and schematically creates an animated blood clot (Visual Clot) containing all hemostatic components necessary to interpret the results in real-time [20,23,26]. Video S2 in the Supplementary Materials shows an example of the Visual Clot.

The Haemostasis Traffic Light is a cognitive aid for simplifying decision making during perioperative bleeding. This structured algorithm helps prioritize therapeutic interventions according to the pathophysiology and the severity of the bleeding using a simple and ubiquitous symbol: the traffic light. The Haemostasis Traffic Light’s content can be adapted to local algorithms [21]. Figure 1 shows the Haemostasis Traffic Light adapted to the University Hospital of Zurich’s coagulation algorithm.

Voice alerts and auditory icons are acoustic alarm modalities that not only attract attention but also convey further information [24,27]. Voice alerts transmit information using clear spoken language. Auditory icons are short sounds that metaphorically convey the events they represent. We designed voice alerts and auditory icons to indicate vital sign deviations (e.g., high or low blood pressure) [24]. Video S3 in the Supplementary Materials gives examples of the studied voice alerts and auditory icons.

### 2.3. Outcomes for Pooled-Data Analysis

The primary outcome measure was decision confidence. Depending on the study, participants indicated their decision confidence on a 4-point Likert scale (from very unconfident to very confident), a continuous scale from zero to 100 (very low to very high confidence), or a binary selection (unconfident or confident). In addition, as potential influencing factors on decision confidence, we measured task performance, workload (measured with NASA TLX), the use of user-centered technology, gender, job (staff physician, resident physician, anesthesia nurse, or medical student), and work experience (in years).

### 2.4. Online Survey

In addition to the quantitative pooled-data analysis, we conducted an online survey to obtain qualitative data. We prepared six statements targeting the potential influencing variables of decision confidence. Participants could either disagree or agree with the statements (scale: strongly disagree, disagree, neutral, agree, and strongly agree).

For the online survey, we contacted nurses and physicians by e-mail from the following five study centers: University Hospital of Zurich and Hirslanden Clinic of Zurich in Switzerland, University Hospital of Frankfurt and University Hospital of Würzburg in Germany, and Hospital Clínic de Barcelona, Spain. We provided participants with a link to the survey conducted through SurveyMonkey (SVMK Inc., San Mateo, California, CA, USA), reminding them to complete the survey after one week.

### 2.5. Statistical Analysis

We analyzed a pooled data set from the eight studies described above. Where necessary, we standardized outcome measures. For example, we considered the outcome decision confidence as a binary value in the pooled-data analysis. Other measures, such as a 4-point Likert scale (from very uncertain to very confident) and a continuous scale from zero to 100 (very low to very high confidence), were converted to binary values.

For descriptive statistics, we show means, standard deviations, medians, and interquartile ranges for continuous data and numbers and percentages for categorical data.

We used mixed logistic-regression models with random intercepts per individual and an additional random intercept for the study to evaluate the impact of potential influencing factors on the binary decision confidence variable while adjusting for confounders. In the first calculation, we analyzed 2580 out of 3782 data points due to missing values in perceived workload. To assess the impact of the missing values in the dataset on the results, we opted for multiple imputation by chained equations in a second step. We used 40 imputations per missing value. After multiple imputation, we ran the same mixed logistic regression models again to compare the impact of the missing values on the results.

To analyze gender-related under- and over-confidence, we used descriptive tables to illustrate the relation between the proportion of correct answers per individual and the proportion of situations where the individual considered him- or herself to have good decision confidence.

We present the results of the online survey as numbers, percentages, and medians with interquartile ranges. Further, we used the one-sample Wilcoxon signed-rank test for one sample to test if the median differed from a neutral rating of each statement.

We used R version 4.0.5 (R Foundation for Statistical Computing, Vienna, Austria) to analyze all data. We created figures using Prism 9 (GraphPad Software Inc., California, CA, USA). A *p*-value of less than 0.05 was determined statistically significant.

## 3. Results

We analyzed 3782 decision confidence data points from 421 subjects across eight different studies. Furthermore, we evaluated the responses of 30 anesthesiologists in the online survey. Table 2 shows study and participant characteristics.

### 3.1. Decision Confidence and Task Performance

**Figure 2:**
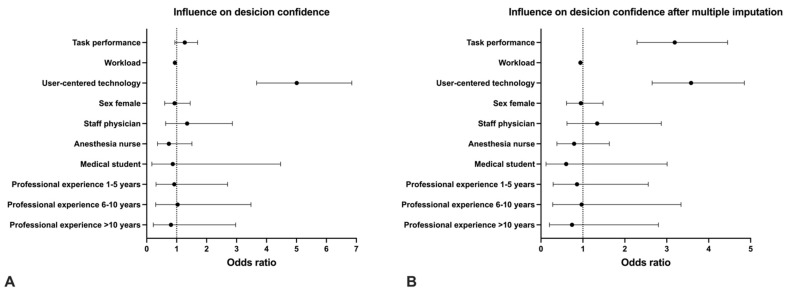
Results for mixed logistic-regression model. (**A**) including 2580 data points of 351 participants and (**B**) after multiple imputation to account for missing values, including all 3782 data points of all 421 participants. Presented bars are odds ratios with 95% confidence intervals.

The mixed logistic-regression model showed that with better task performance, the odds of being confident increased by a factor of 1.27 (95% CI: 0.94 to 1.7, *p* = 0.12). However, this result was not significant. The calculated model is only based on 2580 out of 3782 data points of 351 out of 421 participants due to missing workload (Nasa TLX) values in one study. After multiple imputation accounting for the missing values, the odds of being confident through better task performance increased by a factor of 3.19 (95% CI: 2.29 to 4.45, *p* < 0.001). Figure 2 shows the results before and after multiple imputation.

### 3.2. Decision Confidence and Perceived Workload

Using the same model as above, the odds of being confident decreased by the factor of 0.94 (95% CI: 0.93 to 0.95, *p* < 0.001) with a higher perceived workload, reaching the same outcome after multiple imputation (OR: 0.94, 95% CI: 0.93 to 0.95, *p* < 0.001). Figure 2 shows the results before and after multiple imputation.

### 3.3. Decision Confidence and User-Centered Diagnostic Technology

The mixed logistic regression model also revealed that using a user-centered technology increased the odds of being confident by a factor of 5.01 (95% CI: 3.67 to 6.85, *p* < 0.001). Although after applying multiple imputation, the result showed a slightly smaller yet still statistically significant effect of novel technologies (OR: 3.58, 95% CI: 2.65 to 4.85, *p* < 0.001). Figure 2 shows the results before and after multiple imputation.

### 3.4. Other Potential Influencing Factors on Decision Confidence

We found no evidence for an effect of gender, job, or work experience on decision confidence using a mixed logistic regression model before and after multiple imputation (Figure 2).

### 3.5. Over- and Under-Confidence by Gender

Forty-eight point two percent (95 of 197) of female participants were over-confident, meaning their decision-confidence proportion was bigger than the proportion of correct task performance. Conversely, 30.5% (60 of 197) of female participants were under-confident and 21.3% (42 of 197) provided an appropriate rating. In regards to male participants, we found similar proportions with 50% being over-confident (112 of 224), 29.9% (67 of 224) being under-confident, and 20.1% (45 of 224) were able to assess themselves correctly.

### 3.6. Online Survey

**Figure 3:**
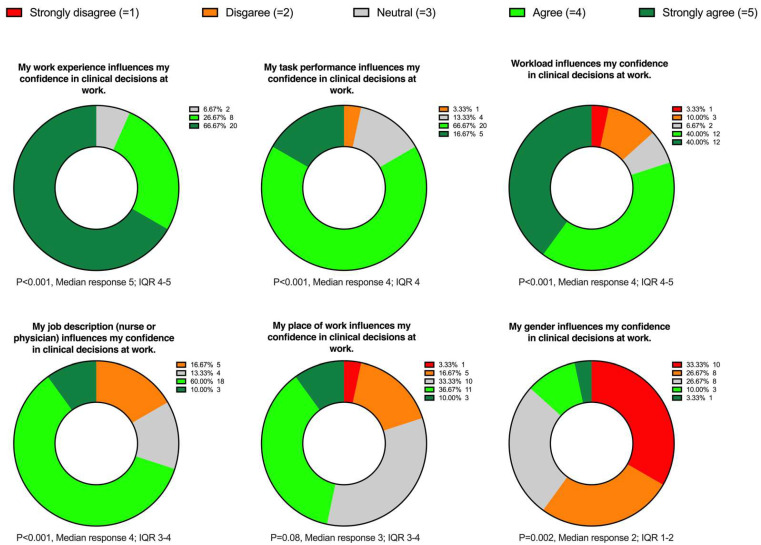
Part-of-whole donut charts display the ratings concerning the six statements of the online survey. Results are shown as numbers and percentages. We used the one-sample Wilcoxon signed-rank test to determine whether the answers differ from neutral (*p* < 0.05). *N* = 30 for each statement.

In February 2022, a sample of the participants from whom the contact details were available was contacted and asked to participate in the online survey on different variables impacting decision confidence. Of the 96 contacted participants, a total of 32 people started the online survey, of which 30 completed it for analysis. Figure 3 illustrates the results of the online survey.

## 4. Discussion

This pooled-data analysis investigated potential influencing factors on decision confidence—the subjective belief to have decided correctly. We found that improved task performance, lower perceived workload, and user-centered technology in medical equipment enhanced the decision confidence of anesthesia providers. However, other factors such as gender, job, and work experience showed no effect in this study. The online survey revealed that over two-thirds of anesthesia staff think that better task performance, lower perceived workload, high work experience, and job influences decision confidence.

Improved task performance results in higher decision confidence in our analysis. This contradicts other studies where no correlation between task performance and confidence could be found [7,8,28]. However, considering that the decision maker has the correct model of the world, it seems logical that their confidence should correspond to their performance. Indeed, some earlier studies on decision making have considered performance and confidence to be similar [2,5] or even interchangeable [29].

In our study, a lower perceived workload resulted in higher decision confidence. This is in line with another study showing a negative correlation between perceived workload and self-rated confidence [16]. However, a common finding in confidence research is the hard-easy effect [30,31]. The effect describes the phenomenon that subjects display greater confidence but lower performance on more complex questions (higher workload) than on easy questions. Despite contradicting our results, whether this effect exists or is due to uncontrolled bias is a subject for further research [30,31]. Another point to consider concerning workload and decision confidence is clear over- or under-challenge. For example, students tend to become inattentive out of boredom during an under-challenging class, whereas a fast-paced, complex lecture can lead to a lack of comprehension. Both can cause poorer performance and probably lower confidence [32].

We found that the user-centered design of medical diagnostic equipment enhanced the decision confidence of anesthesia providers. Such design features incorporate and take into account human cognitive limitations and should simplify and visualize the structure of the tasks so that actions are intuitive at all times. Additionally, it should establish the proper mappings between required actions and intended outcomes [25]. In other words, the goal is to design a system that transmits needed information to the operator as quickly as possible and without undue cognitive effort. In terms of medical devices, this ideally leads to a quick understanding of the situation followed by faster diagnosis and treatment of the patient. Therefore, it is logical that a user-centered design also results in higher decision confidence and lower perceived workload. For this reason, we advocate the increased introduction of medical tools with user-centered designs into everyday medical practice to support healthcare providers in making the right decisions and ensuring optimal patient care.

The online survey showed that participating anesthesiologists agreed that task performance and perceived workload affects decision confidence. Furthermore, the participants rejected gender as an influencing variable. These findings are consistent with the pooled-data analysis. Interestingly, many anesthesiologists stated that work experience, job, and even workplace influence decision-making behavior. This contradicts the results from the pooled-data analysis, which revealed no correlation between the variables mentioned and decision confidence, offering grounds for further research.

### Strength and Limitations

This study has limitations. Data collection occurred in a protected study environment. Solving tasks on a computer or in a high-fidelity simulation differs from diagnosing and treating actual patients, in which wrong decisions potentially can cause harm. It is possible that participants were less reflective of their self-rated decision confidence and workload levels. In addition, all participants were recruited in large tertiary hospitals in Europe and South America. These results may differ elsewhere in the world under different working and socio-cultural circumstances. Nevertheless, we consider the multicenter approach a strength of our study. Another strength is that we chose a mixed-methods study design. We evaluated a large quantitative data pool and followed up with a qualitative survey. Such an approach is often more powerful than quantitative data alone for complex human-factor questions [33]. Further strengths of the pooled-data analysis are the similar study protocols, the consistent recording of identical variables, and the fact that all studies analyzed were initiated by the same study team. Therefore, the studies are readily comparable, a prerequisite for merging study data into a pooled data set [34].

## 5. Conclusions

Better task performance, lower workload, and user-centered design in medical diagnostic equipment enhanced the decision confidence of anesthesia providers. Other factors, such as gender, job, professional experience, or study center, do not affect decision confidence. These results contribute to a deeper understanding of decision making and highlight its importance in the medical field. Furthermore, they pave the way for the further implementation of user-centered designs in medical devices.

## Figures and Tables

**Table 1:** Summary of the eight studies analyzed. Self-assessed confidence was collected in each study after the completion of a specific task using either a new or an established technology. For further analysis, we defined the endpoint of self-assessed confidence of the eight studies as a binary value (confidence below the median versus confidence at or above the median). User-centered technologies were Visual Patient, Visual Clot, Haemostasis Traffic Light, Voice alerts, and auditory icons. USZ: University Hospital of Zurich, Switzerland; KSW: Cantonal Hospital of Winterthur, Switzerland; UKF: University Hospital of Frankfurt, Germany; UKW: University Hospital of Wuerzburg, Germany; BA: Hospital Italiano de Buenos Aires, Argentina; BCN: Hospital Clínic de Barcelona, Spain.

Study	Location	Participants	Tasks Processed Per Participant	Assessed Confidence Levels	Endpoint of Assessed Confidence
Tscholl et al., 2018 [17]	USZ, KSW	32	4	128	Four nominal scale-levels
Garot et al., 2020 [18]	USZ, KSW	39	4	156	Continuous data from 0 to 100
Pfarr et al., 2020 [19]	USZ, KSW	39	4	156	Continuous data from 0 to 100
Rössler et al., 2020 [20]	USZ, UKF	60	12	720	Four nominal scale-levels
Kataife et al., 2020 [21]	USZ, BA	84	6	504	Four nominal scale-levels
Roche et al., 2021 [22]	USZ	104	3	312	Binary
Said et al., 2021 [23]	USZ, KSW, UKF, UKW, BCN	35	18	630	Binary
Roche et al., 2021 [24]	USZ, KSW, UKF, UKW	28	42	1176	Binary

**Table 2:** Study and participant characteristics. Data presented as numbers (%) or median (IQR (range)).

Study Characteristics	Number
**Studies included**,	8
Study centers included	6
Decision confidence data points analyzed	3782
Missing workload data points accounted for by multiple imputation.	1202
**Participant characteristics of pooled-data analysis**	
Participants, *N*	421
Sex female	197 (47%)
Job	
Resident physician	175 (41.6%)
Staff physician	125 (29.7%)
Anesthesia nurse	95 (22.6%)
Medical student	26 (6.1%)
Work experience, in years, *median (IQR (range))*	4 (1–9)
**Participant characteristics of online survey**	
Participants, *N*	30
Sex female	12 (40%)
Job	
Resident physician	6 (20%)
Staff physician	15 (50%)
Anesthesia nurse	9 (30%)
Age in years	34 (30–39)

## Data Availability

Not applicable.

## Supplementary Materials

The following supporting information can be downloaded at: https://www.mdpi.com/article/10.3390/diagnostics12081835/s1, Video S1: Visual Patient Avatar training video; Video S2: Visual Clot training video; Video S3: Example of studied voice alerts and auditory icons.
